# Wilckodontics: A Multidisciplinary Approach for Orthodontic Treatment

**DOI:** 10.7759/cureus.47590

**Published:** 2023-10-24

**Authors:** Mahima Jaswani, Priyanka Jaiswal, Amit Reche

**Affiliations:** 1 Public Health Dentistry, Sharad Pawar Dental College and Hospital, Datta Meghe Institute of Higher Education and Research, Wardha, IND; 2 Periodontics, Sharad Pawar Dental College and Hospital, Datta Meghe Institute of Higher Education and Research, Wardha, IND

**Keywords:** bone grafting, accelerated orthodontic treatment, regional acceleratory phenomenon, corticotomy, periodontally accelerated osteogenic orthodontics (paoo)

## Abstract

Wilckodontics is the periodontally accelerated orthodontic treatment, which is a clinical procedure that combines orthodontic tooth movement with corticotomy and bone grafting. Corticotomy is the surgical procedure that involves cutting the bone, perforating the bone and mechanically altering it. This procedure makes tooth movement easy and rapid with the help of orthodontic force application. This procedure is based on the regional acceleratory phenomenon that increases the bone width, shortens the treatment time from years to months and increases the treatment stability. This procedure also reduces the need for extraction and also increases bone support for teeth and soft tissues. This review article describes the surgical procedure, advantages, disadvantages, indications, and contraindications of Wilckodontics and the current advances in this technique.

## Introduction and background

Orthodontic therapy is necessary to accomplish the orthodontic goals of a functional and aesthetically pleasing dentition, which the majority of patients and professionals prefer. By using either fixed or removable appliances and using various forces, orthodontic tooth movement can be achieved. In today’s time, dental appearance plays an important role in defining the facial features of an individual, boosting one's self-esteem in social interactions [1-4]. Recent studies stated the correlation between dental malocclusion, psycho-social well-being, and self-esteem [5]. There is a hike in the number of patients desiring correction of malaligned teeth and aesthetic dentistry. To achieve the desired dentition, orthodontic tooth movement is required that can be brought about by application of force in a particular, or desired, direction. The duration of orthodontic treatment is approximately one to two years, which completely depends upon the severity of the case and the treatment plan. Longer treatment times lead to a potential risk of developing further dental issues such as root resorption, caries, and periodontal disease because of poor oral hygiene [6-8]. To reduce the duration of orthodontic treatment, due to the demand of the patient and to avoid the adverse effects, orthodontists have tried a variety of techniques to speed up tooth movement. Among them, periodontally accelerated osteogenic orthodontics (PAOO) technique has been the most widely accepted, as it has notably decreased the treatment duration [9-11].

Wilckodontics, also known as PAOO, is a clinical procedure that combines orthodontics with periodontal treatment to accelerate the process of tooth movement. It reduces the appliance-associated discomfort and reduces the treatment time of the orthodontic procedure. This comprises bone grafting and surgical alveolar decortication; decortication is the purposeful cutting of the cortical bone. The long-term benefits of this surgery include a shorter orthodontic treatment course and improved periodontium [12].

## Review

Methodology

**Figure 1:**
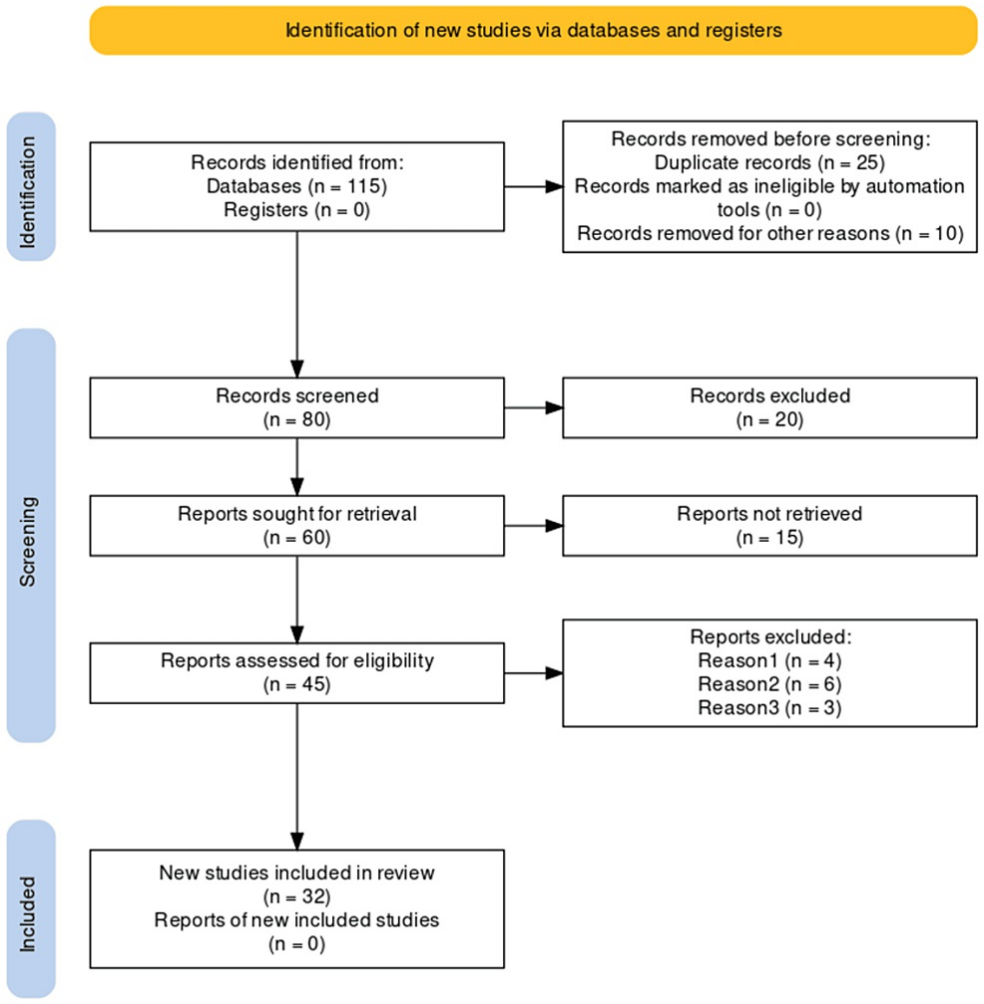
Selection process for articles included in this study Adopted from the Preferred Reporting Items for Systematic Reviews and Meta-Analyses (PRISMA).

We undertook a systematic search through PubMed and Google Scholar in September 2022 using keywords such as "wilckodontics", "regional acceleratory phenomenon", "Periodontally accelerated osteogenic orthodontics", (((wilckodontics[Title/Abstract]) OR ("wilckodontics"[MeSH Terms]),(("regional acceleratory phenomenon"[Title/Abstract]) OR ("regional acceleratory phenomenon"[MeSH Terms]),("Periodontally accelerated osteogenic orthodontics"[Title/Abstract]) OR ("Periodontally accelerated osteogenic orthodontics"[MeSH Terms]). A total 32 articles were included. Figure 1 shows the Preferred Reporting Items for Systematic Reviews and Meta-Analyses (PRISMA) flow diagram for search strategy.

History

Corticotomy-facilitated tooth movement was first described by LC Bryan in 1893 and was published in a textbook by Guilford [13]. Henrich Kole, in 1959, stated that “due to the width and continuity of cortical bone, resistance is caused to the tooth movement”, which resulted in the discovery of “bony block movement”; he proposed that movement of bone blocks, in which the teeth are embedded, was possible by disturbing the cortical bone continuity [14]. In 1975, Düker studied the impact of corticotomy on the tooth and viability and came to the conclusion that “marginal bone should be conserved” and "incisions should be given interdentally 2mm apical to the level of alveolar crest” [15].

Wilcko et al. made further modifications to this approach, by adding corticotomy-assisted orthodontic tooth movement along with alveolar augmentation with the help of the combination of de-mineralized freeze-dried bone allograft/xenograft/absorbable allograft, and named it as periodontally accelerated osteogenic orthodontics [16].

Regional acceleratory phenomenon

Herald Frost, an orthopaedic surgeon, discovered in 1983 that surgical damage of osseous tissue causes a dramatic reorganising activity near the injury site (can be the bone or soft tissue surgery). This series of healing processes was described as the "regional acceleratory phenomenon" (RAP) [17]. In the regional regenerative/remodeling process, RAP is the local reciprocation of the tissue to the unpleasant/painful stimuli that makes tissue regeneration quicker than normal. The response differs in duration, size, and intensity with the varying range of stimuli. RAP generally endures for about four months in human bone, but it depends upon the type of tissue. RAP makes the healing of bone 10 to 50 times faster than the normal bone turnover [18].

Rat tibia has been used as the study model for understanding the healing phases of RAP. The healing phase begins with the initial stage, i.e., the formation of woven bone; it starts in the periosteal area, extending to the medullary bone and attains the utmost thickness on day 7. The fundamental component of RAP is cortical bone as it provides mechanical stability to the bone after injury. In cortical area, woven bone undergoes remodelling to lamellar bone from day 7, but in the medullary area, woven bone undergoes resorption, i.e., transitory local osteopenia.

In long bones of human, after the injury, RAP begins in some days, peaking at about one to two months and taking 6 to 24 months to subside properly [17]. This results in decreased bone density in healthy tissues, but the bone matrix volume does not change. The application of orthodontic force is the adequate stimulant to activate mild RAP activity. When tooth movement is combined with decortication, this leads to the maximum RAP.

Wilcko et al. suggested that an activated bone layer can be applied to the surface of teeth to facilitate tooth movement surgically in the direction required for the movement of tooth. De-mineralization of the thin layer of bone creates a matrix of soft tissue and islands of osteoids that are carried with the root surface of the teeth and are re-mineralized at the desired position. The process of re-mineralization is complete in children but is only partially completed in adults [19].

Indications

Wilckodontics is used in anterior open bite and deviated midline cases; it can also be used in correcting crossbite and tooth discrepancies. This method is beneficial in accelerating canine retraction after premolar extraction. This approach is also helpful in amplifying post-orthodontic stability and speeding up impacted tooth eruption; it also promotes slow orthodontic expansion.

Contraindications

Wilckodontics is not advised for patients having severe periodontal disease, endodontic problems that have not been treated properly and patients with severe class III malocclusion. This procedure is not suitable in patients who are on long-term medications, e.g., non-steroidal anti-inflammatory drugs, and patients on long-term steroid therapy, as it slows the bone metabolism. Patients having any bone metabolic disorders, such as osteoporosis, or any systemic disease are also contraindicated for this procedure.

Surgical procedure

Unlike corticotomy, PAOO just does not cut the osseous structure, but rather decorticates it, i.e. some of the bone's outer surface is reduced. The removal of bone's external surface leads to osteopenia, in which there is a brief drop in the mineral concentration of the bone temporarily. As a result, deposits are released from the alveolar bone by tissues that are rich in calcium; the mineralization of the new bone begins within a span of 20-40 days. This leads to rapid tooth movement with the help of the orthodontic appliance as the alveolar bone is in a transitory state, and has become more pliable and there is less resistance to the force derived from the fixed appliance. Prior to performing the PAOO operation, the anchoring is established for malocclusions that require retraction.

Bracket placement and the activation of the orthodontic wires is usually done one week prior to the corticotomy procedure is performed. The application of orthodontic force should be initiated within two weeks after the corticotomy procedure. The time taken for accelerated tooth movement is approximately four to five months, after which finishing movements occur at a normal speed. Within this period of rapid movement, it is essential for the orthodontist to rapidly advance the arch wire sizes.

Case Selection Criteria

Wilckodontics can be performed in patients belonging to any age group with a healthy periodontium. It is also suitable where conventional fixed orthodontic treatment is done, and in patients with class I malocclusion having crowding that ranges from moderate to severe and class II malocclusion that requires expansion and extraction.

Flap Designing

Combining a full-thickness flap with a split-thickness flap creates the fundamental flap design. The full-thickness flap is raised from both lingual and labial sides in the coronal aspect whereas the split-thickness flap is raised in the apical aspect. Split thickness is used as it provides mobility to the flap so that less tension is applied while suturing. In maxillary central incisors, the interdental papillary area is preserved for aesthetic purposes. In-depth debridement followed by curettage is done following flap reflection [20]. The periosteal layer is removed for better accessibility to the alveolar bone; it also helps in identifying the underlying neurovascular structures.

In anterior teeth cases, tunneling is performed from the distal site. The procedure of decortication is performed with the help of no. 1 and no. 2 round burs and the piezoelectric knife. The groove, which extends up to 2-3mm below the bone crest, is then positioned between the root prominences. The circular corticotomy is then joined to the vertical corticotomy. It is important to take appropriate precautions to avoid damaging the underlying tissues.

Grafting

The areas that have undergone the surgical procedure (corticotomy) require grafting. The draft material volume varies, depending on the amount and direction of tooth movement, thickness of the alveolar bone and the support required for bone [21]. Autogenous bone, deproteinized bovine bone graft, decalcified freeze-dried bone allograft (DFDBA), and combinations of these materials are among the materials utilized for bone grafting. Clindamycin phosphate or bacteriostatic water solution, approximately 5mg per ml, or calcium sulphate or platelet-rich plasma is used to moisten the particulate bone grafting material that facilitates the easy placement of the graft material and increases its stability. Flaps are approximated and non-resorbable sutures are used to close the flaps. The sutures are then removed after one to two weeks [22]. As the time required for epithelial attachment establishment is ideally two weeks, sutures are removed only after the completion of this time period [23].

As a single PAOO treatment procedure can take a few hours to complete if both maxillary and mandibular arches are treated, using short-term steroids improves patient comfort and clinical recovery. Analgesics and antibiotics can also be administered.

Post-surgical Complications

Complications usually include edema and ecchymosis. Antibiotics, analgesics, and application of ice pack to the affected area are advised as they help to reduce the pain and severity of postoperative swelling or edema and infections [24]. Patients are instructed for the post-surgery follow-ups, weekly for the first month and monthly thereafter, for evaluation and prophylaxis.

There are many advantages of Wilckodontics. The procedure shortens the treatment time, prevents injury of the periodontium, prevents pocket formation, prevents devitalization of the tooth, and helps in accelerated tooth movement; the chances of relapse are reduced or decreased drastically and the possibility of resorption of the root is also decreased. In addition, the level of discomfort and irritability is reduced as the movement of tooth occurs due to the softened bone.

However, this treatment is more expensive than the normal procedure and requires additional surgical procedures. There are increased chances of infection, pain or swelling after the surgical procedure.

Certain modifications of this technique have been proposed, which include combining the PAOO procedure with gingival augmentation in patients with severe gingival recession. In these cases, subepithelial connective tissue graft is placed over the denuded root surface. This graft is taken from the elevated flap by removing 1 to 2 mm thickness of the gingival connective tissue [25].

Table 1 shows results of the previous studies conducted in this field.

**Table 1:** Summary of studies performed in the previous years PAOO, periodontally accelerated osteogenic orthodontics; MTDLD, monocortical tooth dislocation and ligament distraction; RAP, regional acceleratory phenomenon

S. no.	Authors	Year	Place	Results
1	Wilcko et al. [1]	2009	USA	Rapid tooth movement was observed and retention and stability were seen up to eight years.
2	Lee et al. [2]	2008	USA	Corticotomy and osteotomy both can be used for tooth movement, but both induce different alveolar bone reactions.
3	Nowzari et al. [3]	2008	USA	In adults, PAOO is the effective procedure for orthodontic treatment that helps in reducing treatment time and decreasing the probability of root resorption, while maintaining the alveolar bone thickness.
4	Sebaoun et al. [4]	2008	USA	Selective alveolar decortication induced increased turnover of alveolar spongiosa.
5	Wang et al. [5]	2009	China	Tooth movement assisted by corticotomy showed bone resorption; after 21 days, it was replaced by fibrous tissue, and by bone after 60 days. Corticotomy-assisted tooth movement was similar to distraction osteogenesis and did not pass through the bone resorption stage.
6	Wilcko et al. [6]	2008	USA	The new approach of rapid tooth movement has made surgical methods easier as it helps in closing the extraction space in 3-4 weeks.
7	Köle [7]	1959	Austria	Corticotomy of maxillary and mandibular bone helps in the displacement of tooth with the help of suitable orthodontic appliances, which reduces the treatment time and the risk of relapse.
8	Düker [8]	1975	Germany	Teeth can be rearranged by corticotomy within a few days when bone is weakened, and vascular supply of pulp is not damaged by rapid tooth movement.
9	Nimeri et al. [9]	2013	USA	Piezocision is considered one of the best techniques to perform as it gives good aesthetic results and goof periodontal tissue response.
10	Wilcko et al. [10]	2001	USA	The degree of re-mineralization and de-mineralization decides what happens in alveolar bone during tooth movement.
11	Mathews and Kokich [11]	2013	USA	In accelerating tooth movement, corticotomy is effective, but it cannot be concluded that it reduces the orthodontic treatment time.
12	Wilcko et al. [12]	2001	USA	This new orthodontic method reduced the treatment time to 6 months (from bracketing to de-bracketing) and reduced bone resorption was noted.
13	Düker [15]	1975	Germany	Corticotomy helps in re-arranging the tooth within a shorter duration of time without affecting the vascular supply of pulp.
14	Vercellotti and Podesta [16]	2007	Italy	As compared to traditional orthodontic therapy, the average treatment time with the MTDLD technique in the maxilla and mandible was reduced by 70% and 60%, respectively.
15	Frost [17]	1989	USA	After RAP also, there is biologic failure of bone formation due to inability to form callus.
16	Schilling et al. [18]	1998	Germany	The model of bone healing revealed that inflammation-mediated osteopenia was not able to inhibit osteoblasts from producing woven bone during RAP or from producing bone that was required to stabilize the defect.
17	Gantes et al. [19]	1990	USA	Corticotomy caused minimum changes in periodontal attachment apparatus.
18	Pavlíková et al. [22]	2011	Czech Republic	Rapid tooth movement reduced treatment time by 60%-70%.
19	Hwei and Thomas [24]	2014	India	PAOO has been proven to reduce orthodontic treatment time, increase bone thickness due to inclusion of bone grafts, provide better post-orthodontic stability and reduced root resorption.
20	Murphy et al. [25]	2009	USA	PAOO results in increased alveolar width, reduced treatment time, and increased post-orthodontic treatment stability.
21	Adusumilli et al. [26]	2014	India	The application of PAOO can be used in borderline dental class III malocclusions; however, it cannot be used for skeletal malocclusion. Age is not considered as a limiting factor as PAOO is the boon for older population, due to its ability to increase the tissue turnover rate two- to threefold.
22	Park [27]	2016	-	Corticision accelerates tooth movement.
23	Alikhani et al. [28]	2013	USA	Micro-osteoperforation reduces treatment time, accelerates tooth movement; it is an effective and a comfortable and safe procedure.
24	Al-Khalifa et al. [29]	2021	Saudi Arabia	Micro-osteoperforation has proven to be a minimally invasive technique for accelerating tooth movement, which has minimal consequences.
25	Dibart et al. [30]	2009	USA	The new minimally invasive procedure allows great patient acceptance, shorter treatment time, stronger periodontium.
26	Abbas et al. [31]	2016	Egypt	Corticotomy-assisted procedure and piezocision are effective treatment modalities for accelerating canine retraction.

Advances

Laser

Corticotomy done with the help of laser is observed as a useful procedure as it is noninvasive. This procedure uses certain elements such as erbium, chromium-doped yttrium, scandium, gallium, and garnet. This procedure is performed without reflecting the flap; hence, the chances of surgical intervention are decreased [26].

Corticision

The procedure of "corticision" was introduced by Park in an effort to present a minimally invasive technique, in place of corticotomies. In this flapless procedure, cortical incisions are placed by separating the inter-proximal cortices transmucosally with the help of a mallet and scalpel [27].

Micro-osteoperforation

Micro-osteoperforation is done using the Propel system, and the technique was introduced by Alikhani et al. [28]. They performed a clinical trial with a sample size of 20 adults having class II division 1 malocclusion. Micro-osteoperforation was first performed on the premolar extraction site. On the buccal surface of the extraction socket, three holes were made that were 5mm away from the alveolar crest. The width of these perforations was 1.5mm and the depth was about 2-3mm. This study showed a reduction in the duration of treatment by 62% and a 2.3-fold increase in the rate of canine retraction when compared to the control group. Micro-osteoperforation is a repeatable surgical procedure, which is effective and less invasive, along with reduced discomfort and pain associated. This procedure has also been shown to increase the production of cytokines and chemokines that help in stimulating osteoclast differentiation and recruiting osteoclast precursors [29].

Piezocision

Piezocision was first described by Dibart et al.; the technique includes peizosurgery without the elevation of the flap [30]. It includes incision at the inter-dental gingiva followed by corticotomy with a piezo-electric device. This method reduces tissue damage as it is a less invasive technique. However, it slows down the tooth movement, when compared to the conventional corticotomy procedure [31]. There are two types of peizocision: conventional peizocision and novel peizocision. In conventional piezocision, piezosurgery is used together with a conventional flap [16], and in novel piezocision, microincision is given to the buccal side with a piezoelectric knife and tunneling for soft and hard tissue grafting [32].

## Conclusions

Wilckodontics is the latest combination of orthodontics and periodontics on the similar bony platform that has made adult orthodontics a reality. This procedure has been made possible with the help of regional acceleratory phenomenon. It increases the bone density due to grafting of the bone and enhances the post-orthodontic stability that decreases the chance of root resorption. The tissue response is found to be beneficial in the treatment of crowding, molar intrusion, etc.

Wilckodontics aims at accelerating the treatment duration unlike the traditional orthodontic treatment, as traditional orthodontic procedures majorly focus on tooth movement only, which consumes a lot of time. It enhances the dental aesthetics and elevates patients' attitudes towards orthodontic therapy. However, the key to the success of this procedure is proper patient selection criteria, diagnosis and treatment planning, and proper co-ordination and communication between the orthodontist and periodontist while proceeding towards the procedure; this would lead to proper treatment with decreased treatment time and lesser damage to the supporting tissues and teeth. Established guidelines could not be found for the selection of osteotomy or corticotomy, but the latter was proven to be advantageous. To conclude, Wilckodontics is a treatment option with a high likelihood of success and is a "win-win" situation for both the patient and the doctor.
